# Object Handling for People With Dementia: A Scoping Review and the Development of Intervention Guidance

**DOI:** 10.1093/geroni/igac043

**Published:** 2022-06-13

**Authors:** Federica D’Andrea, Tom Dening, Victoria Tischler

**Affiliations:** School of Biomedical Sciences, University of West London, London, UK; Mental Health and Clinical Neurosciences, School of Medicine, University of Nottingham, Nottingham, UK; European Centre for Environment and Human Health, Medical School, University of Exeter, Exeter, UK

**Keywords:** Dementia care, Heritage items, Nonpharmacological interventions, Object handling, Psychosocial interventions

## Abstract

**Background and Objectives:**

Among the various psychosocial interventions aiming at improving behavior, quality of life, and the well-being of people with dementia, one that has attracted recent attention has been object handling. This scoping review synthesizes available studies on object handling for people with dementia, their effects, and methodological characteristics and describes its components and likely domains.

**Research Design and Methods:**

The search was conducted using CINAHL, PsycINFO, MEDLINE, PsycARTICLES, Academic Search Elite, and Art Full Text, plus review of reference lists and hand search. Data from the studies included were chattered and reported in narrative form.

**Results:**

Eleven studies were included; of which, 9 described a group intervention and 10 investigated the distinctive value of heritage items. Studies used a mixed-methods or qualitative design and varied in their procedures, including number of sessions and length of intervention. Most studies reported positive effects on well-being, mood, and emotion in those with dementia. Qualitative investigations revealed that the co-construction of an object’s meaning facilitated new learning, social inclusion, and change in attitudes toward dementia. From the review and stakeholder consultations, a definition of object handling is proposed, which includes three components: presenting, receiving, and responding.

**Discussion and Implications:**

The findings suggest that people with dementia may benefit from object handling interventions as a means of improving well-being, mood, and social inclusion. The review highlighted a variety of approaches used and a small number of studies were identified under the term of “object handling.” Further studies are needed to examine the complexity of object handling, its impact within dementia care settings, and that explicitly use the term “object handling.” Given the focus to date on heritage, archive, and museum objects, more studies involving the handling of everyday material objects are needed because these are by definition highly accessible.

**Translational Significance:** An increasing number of studies have used object handling as a psychosocial intervention in dementia care. However, there is inconsistency in how this term is applied. This review explores the literature on object handling and dementia. The evidence suggests that people with dementia may benefit from object handling interventions as a means of improving well-being, mood, and social inclusion. The paper proposes guidance based on an atheoretical model to describe the components of object handling. The findings, definition, and model are recommended for use in future studies of object handling and dementia.

Dementia is an umbrella term, describing a range of syndromes affecting one or usually more cognitive domains that substantially compromise social and/or occupational functioning of older people aged 65 and older and those with younger onset usually between 30 and 65 years of age (Alzheimer’s Association, 2019). To support people with dementia to live as well as possible with the condition, there is increasing interest in the application of psychosocial interventions aiming at improving behavior, cognition, quality of life, and the well-being of those living with dementia. Well-being is a multidimensional construct referring to individual experiences in physical, psychological, and social domains such as positive emotions, mood, sense of purpose, social engagement, life satisfaction, fulfillment, good physical health, and positive functioning (All-Party Parliamentary Group on Arts Health and Wellbeing, 2017). Among psychosocial interventions, object handling has recently become more commonly reported so it is timely to review this growing literature.

Object handling involves several senses, including touch that plays a fundamental role across the life span (Camic, 2010). This sense becomes particularly important in the later stage of life due to deterioration in the senses of sight and hearing (Behrman et al., 2014). Exploring material objects, defined as “physical items that fill our environment throughout our lives that we use, possess, wear, covet, discard and experience in a myriad of ways every day,” (Solway et al., 2016) through touch and other sensory modalities can assist older people to organize and integrate information from different senses, leading to multiple encoding of information processing, which in turn can facilitate new learning (Shams and Seitz, 2008; Paddon et al., 2014). For instance, it has been suggested that the combination of handling, looking at, and talking about objects may enhance “dual” or even “triple coding” effects (Solway et al., 2016). Theoretical memory models (Craik and Lockhart, 1972; Clark and Paivio, 1991; Lockhart and Craik, 1990) suggest that when verbal, touch, and visual sensory information are presented together, the items of information become connected with each other in short-term memory (i.e., working memories) during the encoding and become integrated with previous experiences and knowledge from long-term memory. This process results in a deeper elaboration (or cognitive processing) of the physical material information that leads to more connections being laid down in memory. These views have been supported by neuroscientific studies that found that older people (without a diagnosis of dementia) benefit more from receiving multimodal stimulation compared with unimodal stimulation, in performing tasks such as detection or judgment (de Dieuleveult et al., 2017). Indeed, a sensory-enriched experience enables stimuli to be encoded into multisensory representations thereby activating a wider network of brain regions compared with those invoked by unisensory encoding, and thus facilitating older people in performing tasks (de Dieuleveult et al., 2017; Lehmann and Murray, 2005; Matusz et al., 2017) and also compensating for a decline or loss of a unisensory modality (Peter et al., 2019).

Giachritsis (2008) argued that touch could be considered as the “ultimate sense” that enables us to create a complete representation of the world. The mechanisms involved in tactile object analysis and the anatomical correlates of those mechanisms are still poorly understood. Tactile object recognition is likely a priori to involve a number of stages including the initial encoding of elementary sensory data, the integration of sensory information to form a coherent tactile representation of the object, and the association of that tactile representation with semantic knowledge about the object (Crutch et al., 2005). Neuropsychological evidence put forward by Critchley (2008) suggested that there is a close relationship between touch and emotional and motivational systems in the brain, which could explain the sense of well-being that may be evoked through touch. Reflecting these important neurological and functional aspects, objects such as sensory cushions or muffs (also known as “twiddle muffs”) made with soft fabric, buttons, zips, and beads have been widely used with people with dementia.

Lanceley et al. (2012) state that material objects can act as “a repository or container for projections of different and difficult states of mind.” For instance, a growing number of care homes use dolls to comfort residents, drawing on evidence for the benefits of “doll therapy” (Ng et al., 2017). The use of material objects is also central to reminiscence and occupational therapy, aiming to stimulate memories and to enhance independence in daily life, respectively. Rowlands (2008) suggested that older people with dementia benefit from handling familiar objects because the objects have the potential to prompt memories, restore life histories, and express the identities of individuals.

More recently, increasing evidence supports the value of using material objects in health care. Much of this assesses the impact of items from museum and gallery collections, a practice defined here as heritage object handling. Findings from heritage object handling sessions show that manipulating and discussing heritage items can increase participants’ well-being, social inclusion, provide intellectual stimulation, and prompt memories as well as creating links to the present (Camic et al., 2019; Chatterjee et al., 2008; Paddon et al., 2014; Solway et al., 2016; Thomson and Chatterjee, 2016a).

Despite the growth in use of object handling in dementia care, there is no clear definition of what object handling is. There are however some elements common to the practice. This includes offering or choosing a material object, and participants having the opportunity to explore, reflect, and respond to it. This therefore excludes activities like pet or doll therapy where the aims are somewhat different, for example, an intention to modify behavior. The use of a wide range of objects, that is not just heritage items, could also be included. These may incorporate everyday items or other types of curio or memorabilia. These elements inform our working definition of object handling.

The aim of this review is to provide an overview of available object handling interventions and to map the outcomes relating to the impact of the intervention on people living with dementia. It is anticipated that this will help to define what constitutes object handling in dementia care settings.

## Method

A scoping review approach has been selected as the most suitable synthesis method for the current research, as it incorporates a range of study designs and addresses questions beyond those related to treatment efficacy (Lockwood et al., 2019; Peters et al., 2021a). The scoping review was guided by Arksey and O’Malley’s methodological framework (Arksey and O’Malley, 2005) that was further refined by Peters et al. (2015). The framework includes defining the research questions; identifying the eligibility criteria and the research strategy; searching for relevant studies; selecting studies; charting the results; and collating, summarizing, and reporting the results.

The inclusion criteria and methods for the review were prespecified and are presented below according to the Preferred Reporting Items for Systematic Reviews and Meta-analyses Extension for Scoping Reviews (PRISMA-ScR) checklist (Tricco et al., 2018).

### Eligibility Criteria

#### Inclusion criteria

Studies were included if they were written in English and where participants were considered by authors as having dementia even if specific diagnoses were not provided. No specific restrictions regarding geographical, time limits on the publication, age, subtype, and severity of dementia were applied.

In the absence of an agreed definition of object handling, an operational definition was created for the purposes of the review by identifying some similarities in the implementation across studies. The definition of an object handling intervention was as follows. It consisted of a program based on offering or choosing an object, with participants having the opportunity to explore, reflect, and respond to it, before moving to another item. The object(s) used could be of any type, from everyday items to museum artifacts. Group or individual sessions were included. Studies were included if object handling was combined with another activity (e.g., art viewing).

#### Exclusion criteria

Articles describing interventions for use solely by caregivers were excluded. Studies were also excluded if they did not meet the above definition of object handling. These included studies that focused on cognitive training, doll therapy, reminiscence therapy, occupational therapy, Montessori-based activities, art making, and art viewing. Unpublished papers, study protocols, dissertations, and websites were also excluded.

### Search Strategy

The review search was conducted in November 2018 and updated in February 2022 to ensure that all relevant articles were included in the review. The studies were identified using a combination of key terms and databases (Supplementary Table 1). The search strategy was developed with advice from a specialist health librarian.

### Selection of Sources of Evidence

Electronic search results were downloaded into New RefWorks, a reference management software. The lead author screened titles and abstracts identified by the electronic search and applied the selection criteria to potentially relevant papers. Titles for which an abstract was not available were included for subsequent review of the full article. For articles that could not be obtained through institutional holdings, attempts were made to contact the source author to procure the article.

The full text of potentially eligible studies was read independently by the lead author and a second reviewer to assess eligibility. Some study authors were contacted asking for information or clarification if needed. Studies were excluded during this phase if they were found to not meet the eligibility criteria. Any uncertainties related to study selected during the screening process were resolved through discussion between reviewers.

### Data Charting Process

Data were extracted by the lead author using a checklist including authors, country, study design, study aim(s), participant demographic characteristics (i.e., age, types, and stage of dementia), intervention frequency and duration, materials, setting, outcome measures, and relevant outcomes related to the study aim(s). Furthermore, the terms and protocols used to refer to object handling intervention were extracted for each included article. Data charting process was verified by a second reviewer who checked the accuracy of the data on a random 30% sample of studies.

The methodological quality appraisal of the studies included in the review was not conducted given that the focus of a scoping review is to provide an overview of the existing literature. This is consistent with the methodological framework used and standards for scoping reviews (Arksey and O’Malley, 2005; Peters et al., 2015, 2020, 2021b).

### Synthesis of the Results

Studies included were presented in a narrative format in relation to the objectives of the review (Arksey and O’Malley 2005). The details of the study methods and procedures were grouped under main categories including participants’ characteristics, settings, intervention protocol and materials, study design, and outcome measures (Peters et al., 2020). Key outcomes reported by the studies were also included in the synthesis with the scope to determine the range of evidence associated with object handling interventions (Peters et al., 2015). A summary of the relevant study data is also provided in a tabular form.

## Results

### Sample

**Figure 1.**
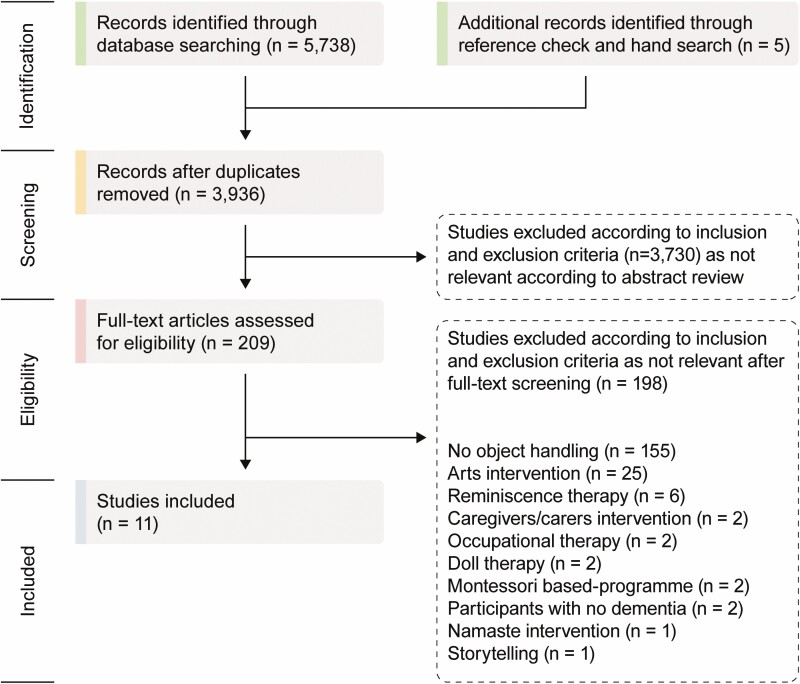
PRISMA flow diagram of scoping review of object handling interventions.

The literature search identified 5,738 articles. Duplicate articles were removed (*n* = 1,964), and inclusion and exclusion criteria applied. Based on titles and abstracts, 209 studies were selected to be further assessed for eligibility. A total of 11 articles (including 2 additions following hand search and reference check) were identified as meeting the inclusion criteria. These included five studies solely on object handling and six articles combining object handling with other activities that, despite the eligibility criteria focusing primarily on handling objects, was deemed by consensus to warrant inclusion because of the very few studies that assess object handling interventions and the overall aim to map the existing literature. Those studies involving multiple activities were included if their aim was not clearly one of the interventions listed in the exclusion criteria (e.g., reminiscence, art making). An overview of the selection process can be seen in Figure 1. All 11 studies were from Europe and varied in design, methodology, number of participants, and measures (see Supplementary Table 2).

### Participants

Across the 11 studies, participants were recruited from a variety of settings, including hospitals, community-based centers, and care homes. Sample size ranged from 2 to 158 participants aged between 62 and 94. Only seven studies reported the level of dementia severity: six included people with mild-to-moderate dementia (Camic et al., 2019, 2021; Griffiths et al., 2019; Hendriks et al., 2018; Johnson et al., 2017; Thomson et al., 2018), and only one study included those with advanced dementia (Norberg et al., 2003). Five studies (Camic et al., 2019, 2021; Hendriks et al., 2018; Innes et al., 2021; Johnson et al., 2017) reported the types of dementia. Griffiths et al. (2019) and Thomson et al. (2018) did not report the type of dementia diagnosis; Innes et al. (2021) did not provide information on the stage of dementia, whereas other two studies (Ander et al., 2013; Thomson et al., 2012) reported neither the stage and type of dementia. It is important to note that Roe et al. (2016) and Thomson et al. (2018) did not include the precise number of people living with dementia who took part in their study. Roe et al. (2016) recruited participants for the museum and gallery program from care homes (*n* = 8) and supporting living facilities (*n* = 9). The authors did not provide information about the participants having a confirmed diagnosis of dementia as they were attending the intervention as “citizens or members of the public” (Roe et al., 2016). Thomson et al. (2018) reported having included people with mild-to-moderate dementia, without disclosing further details. The choice of including Thomson et al. (2018) and Roe et al. (2016) was to provide an overview of the available studies.

As well as people with dementia, six studies included informal caregivers (Johnson et al., 2017; Innes et al., 2021; Roe et al., 2016), health and care professionals (Ander et al., 2013; Griffiths et al., 2019; Roe et al., 2016), and other participant groups, such as neurological rehabilitation and oncology inpatients as well as outpatients (Ander et al., 2013; Thomson et al., 2012). The sample characteristics are summarized in Supplementary Table 2.

### Settings

The studies took place in different settings. Six studies were delivered on museum and gallery sites (Camic et al., 2021; Hendriks et al., 2018; Innes et al., 2021; Johnson et al., 2017; Roe et al., 2016; Thomson et al., 2018), two in care homes (Griffiths et al., 2019; Norberg et al., 2003), one combined sessions in a day center and at a museum (Camic et al., 2019), one study took place in a hospital and care home (Thomson et al., 2012), and one in a health care setting (Ander et al., 2013).

### Procedure and Materials

All interventions were group based, apart from three studies (Innes et al., 2021; Norberg et al., 2003; Thomson et al., 2012) that used one-to-one sessions and one study (Ander et al., 2013) that delivered both group and one-to-one sessions. The interventions ranged from 1 to 16 sessions for a period of 1 week to 6 months. The time for each session ranged from 20 min to 2.5 hr. Most of the studies incorporated object handling session(s) with various activities such as museum and gallery visits, music, massage, art viewing, and art making.

Roe et al. (2016) evaluated a museum event, namely “Coffee, Cake and Culture,” considered as an art program. During this program, participants were invited to “engage in a variety of sensory experiences” through a range of activities that integrated handling and discussing objects, as well as artifacts with storytelling and art making based upon the museum and gallery collection (Roe et al., 2016). Innes et al. (2021) similarly engaged participants with heritage collections alongside storytelling and museum tours.

One multisensory stimulation intervention (Griffiths et al., 2019) combined handling and discussing archival objects with olfactory stimulation. During the sessions, objects were introduced and passed around by participants, who were encouraged to engage with and discuss them. Similarly, a “museum object handling” study (Camic et al., 2021) included material objects and four spices from the museum’s collection with other items such as a prosthetic hand or knitted woollen neurons that were passed one at a time around the group, while the facilitator encouraged touching objects and interactions through prompts and questions.

In Thomson et al.’s (2018) and Hendriks et al.’s (2018) “museum-based social prescription” and “interactive museum programme,” respectively, handling and discussing heritage objects were combined with museum visits and arts activities. Museum-based social prescription is a type of social prescribing that refers to creative activity prescribed by health and social care professionals, often a general practitioner, to address needs such as chronic health problems or loneliness (Drinkwater et al., 2019).

Object handling, massage, and music (e.g., religious and popular songs) sessions were integrated and administered in a systematic way in Norberg et al.’s (2003) study. Objects were introduced to participants one at a time, while the researcher was talking about them.

In the object handling session delivered by Johnson et al. (2017), objects were presented and passed around the group one at a time, giving the participants the opportunity to explore, share personal associations and comments on the physical properties of the items. To encourage individual and group engagement, facilitators asked questions about participants’ experience as they handled and observed the items.

A similar object handling protocol was used in four other studies (Ander et al., 2013; Camic et al., 2019, 2021; Thomson et al., 2012). Camic et al. (2019) placed the emphasis on the importance of using “nonmemory-related” prompts in order to move away from the reminiscence approach and focus on “in the moment” experiences, for example, “Would you have this as a decoration in your home?” or “How does this object make you feel?”. In Thomson et al.’s (2012) protocol, participants were asked to choose the first item to explore and to explain their choice. Prompts focusing on the emotional and physical properties of the items rather than on participants’ autobiographical memories were also used in this study, including stimulating questions such as “What do you think it feels like?” or “How does it make you feel?”.

Table 1 shows the terminology and describes the object handling procedures used in the studies mentioned. There are clearly some similarities between these descriptions. Consistent with the definition of object handling provided in the eligibility criteria, the object handling procedure comprised introducing the objects and giving participants the opportunity to explore and engage with them on different levels (e.g., physical, emotional, meaning, and historical features). Several studies used prompts to promote conversations, as described above.

**Table 1.** Object Handling Procedure of the Studies Included in the Review

Article	Terminology	Procedure
Camic et al. (2021) United Kingdom	Museum object handling	Facilitator passed around “one object at a time and encouraged touching and generating discussion through asking a range of sample prompts and questions to encourage participation and exploration before sharing information about each object. Questions were used flexibly in an open, dialogic way within the sessions based on the interaction of participants. […] Six to nine objects were used in each session.”
Innes et al. (2021) United Kingdom	Heritage program	The program is “designed to provide authentic, creative, site-specific, multi-sensory experience focussing on the ‘here and now’ experiences in a safe dementia-aware environment. The programme’s “Three S’s” model combines sensory stimulation, storytelling (based upon historical information) and period spaces exploration. Individual sessions are designed and delivered by creative facilitators, representing a range of artistic disciplines including sculpture, dance and music, who work to a detailed brief but are given considerable creative freedom in choosing aspects of the site’s ‘story’ to develop their ideas.”
Camic et al. (2019) United Kingdom	Object handling OR museum object handling (when refer to museum)	Objects were “presented to the group, shown to all members without first informing them about the function or name of the object. The object was then handed from member to member so that each individual was given time to have a tactile experience with the object and to have a closer look. As the object was passed around, the facilitator asked a series of non-memory-related questions.” “As each member of the group shared their feelings and opinions, the facilitator encouraged participants to speak more about their responses while holding the objects. When each object made a circuit around the group, it was placed in the centre of the table for all to continue to view.”
Griffiths et al. (2019) United Kingdom	Multisensory intervention	“During the sessions, the facilitator introduced the box and passed round the items. Whilst objects were handled, the facilitator asked questions about the contents and encouraged conversation between the participants. This continued until all the items had been examined.”
Hendriks et al. (2018) The Netherlands	Museum tour	“For each tour, four to six different art works are selected around the theme and presented.” “The guide asks open questions (e.g., about the colors, aesthetic preferences), stimulates interaction between the people with dementia and their caregivers and between the participants, and gives small assignments to be executed in couples, including drawing assignments, to “adopt the same pose as a figure in the artwork” or “talk about the object in couples.”
Thomson et al. (2018) United Kingdom	Museum-based social prescription	“Programmes of engaging, creative and socially interactive sessions, […] comprising curator talks, behind-the-scenes tours, object handling and discussion, and arts activities inspired by the exhibits.”
Johnson et al. (2017) United Kingdom	Object handling OR museum object handling (when refer to museum)	“Object handling sessions comprise tactile, visual, and conversational exploration of authentic museum artefacts.” “Objects were presented one at a time and people had the opportunity to hold, examine, and talk about them as a group as they were passed round. Questions about impressions of the objects included sensory descriptions, preferences, and reflections; associations and anecdotes were encouraged.”
Roe et al. (2016) United Kingdom	Art program	“Over the course of the programme various activities occurred with varying levels of engagement. […] the participants were able engage in a variety of sensory experiences through the diverse range of activities. Visitors looked at or handled a variety of objects, in particular contexts, read exhibition materials, discussed them and took home materials to read and show others.”
Ander et al. (2013) United Kingdom	Museum object handling	Handling session “provided opportunities for learning and discussion about the history and use of these objects [museum objects]”. “Questions were phrased to encourage touching and exploration of the objects.”
Thomson et al. (2012) United Kingdom	Museum object handling	“Participants were invited to choose their first museum object/photograph and suggest reasons for their choice.” The questions that “followed prompted discussion related to the physical and emotional properties of each object/photograph in turn […]. Facilitators referred to fact sheets to address specific questions.”
Norberg et al. (2003) Sweden	Object presentation	“Object presentation consisted of trials to stimulate the patients auditorily, tactilely, olfactorily and visually. The patients were allowed to smell, touch and watch objects and the researcher talked about them.”

Ten studies investigated the distinctive value of heritage object handling for people with dementia; of which, three (Ander et al., 2013; Griffiths et al., 2019; Thomson et al., 2012) used a box to present items to the participants (each containing an eclectic range of objects such as a tiger’s skull, fossilized seaweed, Victorian candle snuffer, Islamic porcelain, old shavers, and infant feeding bottles). Only three studies (Camic et al., 2021; Griffiths et al., 2019; Norberg et al., 2003) included everyday items such as wood, hay, soft soap, yarn, cloves, cinnamon, and knitted woollen items.

Three of 10 studies reported how the objects were selected: based on their tactile, visual and kinesthetic properties (Thomson et al., 2012), on the unfamiliarity and unusual physical features of the items (Camic et al., 2019), or cultural, historical, and sensory qualities (Camic et al., 2021). A list of material objects used in each of the included studies is provided in Supplementary Table 3.

### Study Design and Outcome Measures

Among the 11 studies included in the review, 5 used a quasiexperimental design (Camic et al., 2019; Hendriks et al., 2018; Johnson et al., 2017; Thomson et al., 2012, 2018), which aims to test a causal hypothesis but does not involve randomization. Three studies used a qualitative design (Ander et al., 2013; Griffiths et al., 2019; Roe et al., 2016), two were a mixed-methods design that combines both qualitative and qualitative approach (Camic et al., 2021; Innes et al., 2021), and one used a case study (Norberg et al., 2003).

Four studies used interviews to gather data (Ander et al., 2013; Griffiths et al., 2019; Innes et al., 2021; Roe et al., 2016), visual analog scales were used in four studies (Camic et al., 2019, 2021; Johnson et al., 2017; Thomson et al., 2012), five used observation (Ander et al., 2013; Camic et al., 2021; Hendriks et al., 2018; Norberg et al., 2003; Roe et al., 2016), and three studies used questionnaires along with other measurement tools (Hendriks et al., 2018; Innes et al., 2021; Johnson et al., 2017). Thomson et al. (2018) used the Museum Wellbeing Measure for Older Adults (MWM-OA) (Thomson and Chatterjee, 2015, 2016b), a scale assessing well-being following museum and object handling interventions designed to be administered to older people. Only one study (Norberg et al., 2003) examined physical reactions, recording movements of the eyelids, mouth, and head, as well as physiological reactions, such as pulse and respiration rate, but it did not specify the methods that were used to measure these.

Most of the studies (*n* = 7) investigated the impact of object handling session(s) on the well-being either of participants with dementia or the caregivers supporting them. Five of those studies focused on the participants’ subjective well-being (Camic et al., 2019, 2021; Johnson et al., 2017; Roe et al., 2016; Thomson et al., 2012). Camic et al. (2021) also explored the process underpinning the increase of subject well-being following object handling group sessions (All-Party Parliamentary Group on Arts Health and Wellbeing, 2017).Those studies assessing subjective well-being did so by means of self-report measures, such as visual analogue scales, which were used in four studies to provide ratings of key aspects of subjective well-being: wellness, happiness, interestedness, confidence, and optimism.

Ander et al. (2013) explored the individual experience, emotions, and feelings in relation to the object handling session(s). The remaining three studies focused on the impact of different types of stimuli and stimulation, such as engagement and emotion (Griffiths et al., 2019; Hendriks et al., 2018) and physical responses (Norberg et al., 2003).

### Outcomes for People With Dementia

Six of the studies documented well-being impacts for participants with dementia (Camic et al., 2019, 2021; Johnson et al., 2017; Roe et al., 2016; Thomson et al., 2012, 2018).

Johnson et al. (2017) examined the effect of object handling in comparison to art viewing and to a period of social activity in the form of a refreshment break (involving consumption of food and drinks). The results indicated increased well-being following object handling (*p* < .002) and art viewing (*p* < .006) sessions but not after the social refreshment break. This effect was higher following object handling intervention than art viewing. Data from feedback forms showed that most participants (55%) preferred discussing and manipulating heritage objects compared with the art discussion session (36%).

Thomson et al. (2012) found that handling objects significantly increased the positive mood (*p* < .001), wellness (*p* < .01), and happiness (*p* < .003) of participants compared with sessions based on looking at the same object presented as a photograph, as indicated by improved scores on visual analogue scales (EuroQol Group, 1990). Although the findings showed a decrease of negative mood scores, there was no significant difference between the 2 conditions.

“Beautiful” and “gorgeous” were a few participants’ comments on the objects handled in the program described by Roe et al. (2016). The sessions stimulated positive and enjoyable feelings, promoting increased well-being of those living with dementia.

In line with this finding, Camic et al. (2019) reported a statistically significant increase (*p* < .001) in well-being in participants with early and moderate dementia who participated in object handling in both a museum and day center. The intervention benefits were significantly larger in younger participants (*p* < .03) and in those with early-stage dementia (*p* < .007). No gender differences were found in the well-being score.

Significant positive change between pre- and postsession in the total well-being scores (*p* < .001) was also found in Thomson et al.’s (2018) study. Quantitative analysis showed that two items of the MWM-OA, “enlightened” and “absorbed,” were rated higher than the other four emotions (active, cheerful, encouraged, and inspired) after each session (e.g., smallest increase postsession *p* < .026). When interviewed, participants commented that they felt absorbed while learning new information and skills during the session.

An overall increase in subjective well-being was found following each session in Camic et al.’s (2021) study. However, a significant change was reported in only one subscale (Interested/Bored) of the Canterbury Wellbeing Scale (Strohmaier et al., 2021). Qualitative findings suggested that the facilitators have a central role in creating an atmosphere that supports and promotes exploration of objects from different perspectives as well as active participation. Active participation includes confidence in exploring and discovering more about the objects by discussing and asking questions about the objects to the facilitators and other group members. This led to a sense of group collaboration and cohesion.

Enjoyment, increasing positive emotion, and vitality were reported by Ander et al. (2013) as key themes. These results were linked to an increasing sense of social inclusion and identity derived from the interaction between participant, museum collections, and the group. Participants referred to looking forward to the sessions and they were positive about the nondirective approach of the sessions: “you can choose how much…and what you want to do or say” (Ander et al., 2013).

Although the qualitative study of Griffiths et al. (2019), exploring a multisensory stimulation intervention using selected items from an archive collection, did not directly measure well-being, the authors reported high engagement in all participants, regardless of the level of cognitive decline, and a positive effect on mood.

Hendriks et al. (2018) found statistical differences between engagement with the activity and interactions with other group members according to the severity and types of dementia. People with mild dementia or those with a diagnosis of VaD were more responsive and interactive compared with those with moderate dementia and Alzheimer or other types of dementia. The authors proposed an explanation for this difference, suggesting that people with mild dementia and VaD may retrieve more personal memories, which might directly affect levels of engagement with the session. Moreover, the analysis of the association between responsiveness and specific types or features of items used showed that objects, such as historical items or crockery, were more engaging than artworks.

A case study (Norberg et al., 2003) compared the effect of music, massage, and object presentation in two persons in an advanced stage of dementia. Physical reactions, such as lower frequency of eye blinking and higher verbal reactions, were observed during music stimulation. Both participants did not show any specific physical response to massage and object presentation. According to the authors, a possible explanation of the findings is that participants were not able to perceive the objects due to major sensory impairments.

### Outcomes for Care Professionals and Informal or Family Caregivers

A total of five studies involving care professionals, other staff, and/or facilitators (Ander et al., 2013; Griffiths et al., 2019; Roe et al., 2016) and informal or family caregivers for people with dementia (Innes et al., 2021; Johnson et al., 2017; Roe et al., 2016) explored whether attending object handling sessions in a supportive role had positive effects for them.

Johnson et al. (2017) invited caregivers to complete the same visual analogue-based Canterbury Wellbeing Scales (Camic et al., 2020) as the participants with dementia, and their quantitative analysis of these scores showed a significant increase in subjective well-being after object handling (*p* < .003). Overall, qualitative data indicated that staff and caregiver participants enjoyed the sessions, for example, Innes et al. (2021) found that all caregivers reported to have enjoyed at least one aspect of each session; Griffiths et al. (2019) quoted staff using words such as “lovely,” “emotional,” “uplifting,” “warm and welcoming.” Exploring heritage items was found to be stimulating and to enhance new learning.

Care professionals working in dementia settings reflected on how objects became the main topic in conversations. Participants found that talking about objects was a valuable way to stimulate and engage people with dementia. Roe et al. (2016) found that objects facilitated meaningful discussion between staff and care home residents, shifting the focus from dementia and the caring relationship to broader, nonclinical subjects, thus helping to build and sustain relationships. Care professionals reported that seeing the person in a social context helped to change the way they thought about “dementia” and enabled them to create relationships focused on the person rather than on their disease and disabilities. When interviewed, facilitators noted that mutual engagement and co-construction of the meaning of objects contributed to decreasing power imbalances and hierarchies between facilitators, care professionals, and people with dementia. For instance, some facilitators commented on the process of knowledge exchange and how they learnt from the residents (Roe et al., 2016); others underlined how staff became involved on “an equal footing” to the residents with dementia through the sessions (Griffiths et al., 2019).

## Discussion

This scoping review included 11 studies focused on assessing the impact of object handling interventions in relation to mainly subjective and psychological well-being and emotional response. Other outcomes such as physical response, engagement, and interactions were investigated as well. Most studies used qualitative or quasiexperimental designs. These can offer insights into the most effective features of object handling and capture the benefits of object handling at an individual level and in relation to social interactions “in the moment.”

Most of the studies on object handling placed the emphasis on using objects as tools to create a space where participants could engage and connect with the items and other participants “here and now.” Moving beyond reminiscence and shifting the focus from the past to the present experience of people with dementia recognizes the value of being in the moment. The concept of “in the moment” related to people with dementia has been recently defined as “a relational, embodied and multi-sensory human experience” embedded with personal value, significance, and meaning (Keady et al., 2022). This definition highlights that, for a person living with dementia, moments can be initiated both by themselves, through the recall and response of a particular stimuli, or from interpersonal interaction with other people.

Some evidence suggests that object handling interventions are associated with increased well-being, positive emotion, and social inclusion in participants with dementia, and can help to facilitate new learning and meaningful conversation for caregivers and people with dementia. However, the scoping review was simply concerned with describing the studies and the observed outcomes. Attempting a synthesis of their effectiveness was beyond its aim.

Included studies encompassed different settings and varied greatly in terms of factors such as number of sessions and length of intervention. Most studies were delivered in museum and art gallery venues. This is in line with numerous studies and Camic and Chatterjee’s “cultural and health framework” that suggest offering health care interventions within cultural heritage organizations could promote health and well-being, for example, reducing social isolation, boosting the sense of connection, and belonging by offering nonstigmatizing and nonclinical activities within communities (Camic and Chatterjee 2013). Some studies in the review included other activities such as art viewing and art making alongside object handling. Moreover, there was no clear indication if there is an optimum number of sessions or an optimum duration of interventions. On the one hand, comments were recorded at interview about the cumulative benefits of regular sessions, yet studies that offered only a single session (Camic et al., 2019; Hendriks et al., 2018; Johnson et al., 2017; Thomson et al., 2012) reported similar benefits to others with more sessions.

Seven of 10 studies reported the participants’ stage of dementia. Mainly, people with mild-to-moderate dementia were included and only one study involved those with severe dementia, so there is currently limited evidence as to what extent object handling may be useful for people with advanced dementia.

Regarding the materials used, most of the studies incorporated museum, archive, and heritage objects. Given the historical and unusual features of museum, gallery, archive, and heritage objects, they are likely to have stimulated curiosity or to have connected with the earlier years of participants’ lives. One feature of studies using heritage and museum collections (Camic et al., 2019, 2021; Griffiths et al., 2019) was the sometimes ambiguous nature of the objects; it appears that this may be important in capturing participants’ attention, curiosity, engagement and providing them with a cognitive challenge. It has been argued that curiosity is closely related to well-being as it can drive new learning, creativity, and social connection, each of which can be both a form of and a pathway to well-being (Kador and Chatterjee, 2021; Phillips et al., 2015). The results of this review, therefore, offer support that museum, archive, and heritage object handling interventions have a positive impact on people with mild-to-moderate levels of impairment. It may be desirable that the selection of material objects is tailored to the cognitive and communication impairments of people living with dementia as these could affect how people engage, interact with, and respond to the items. For instance, people in the mild and moderate stages of dementia might benefit more from engaging with archive, heritage, and museum material objects because they are intrinsically interesting and present unusual physical and material properties that could promote social interaction, meaning-making, and new learning opportunities. Whereas when designing object handling interventions for people living with late-stage dementia, familiar or everyday material objects that can provide opportunities for enriching sensory and kinesthetic experiences and necessitate less discussion, such as textiles, or olfactory items, would be more appropriate (Treadaway et al., 2019). Additional research is warranted to further explore what different types of material objects (Fleetwood-Smith et al., 2022) may be able to offer across the stages of dementia.

This review provides an overview of the empirical evidence available on object handling interventions and reveals considerable variety of design and procedures used in the studies. Such heterogeneity may be due to a lack of definition of what an object handling intervention is. Therefore, from the review, the initial concept of object handing was reviewed, and a theoretical understanding of the components and likely domains of action of object handling was developed by drawing on the results of the scoping review, existing theories and evidence, as well as consulting with health and social care professionals, and dementia specialists.

### Components and Domains of Object Handling Intervention

**Figure 2.**
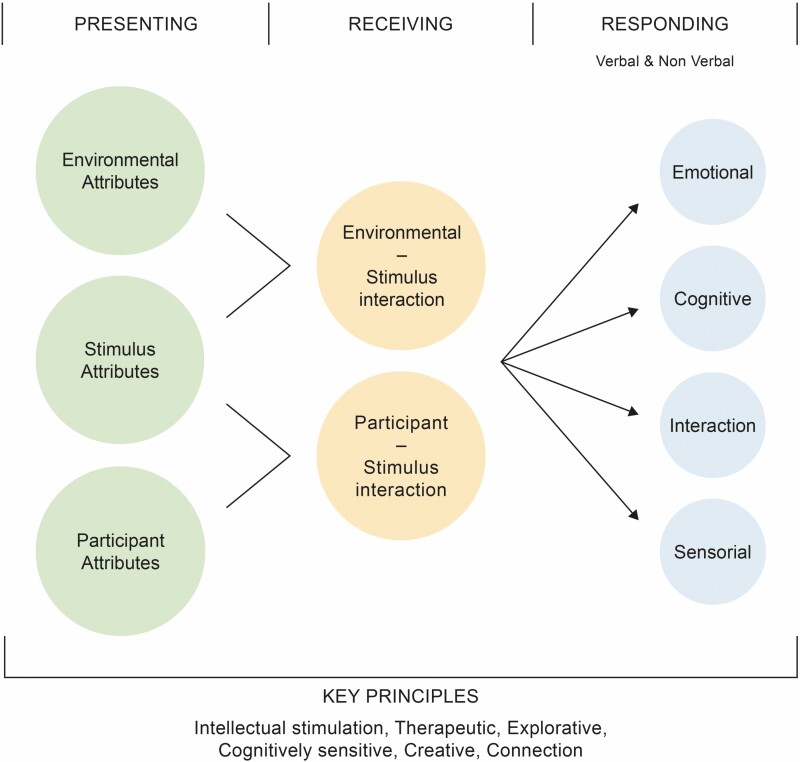
The components and likely domains of object handling.

The results from the literature and stakeholders’ feedback enabled the generation of a list of components and domains, which fed into the development of guidance for object handling intervention (Figure 2). It is suggested that object handling has the following essential components: presenting, receiving, and responding. Furthermore, object handling intervention may be conveyed in terms of a set of underlying factors (principles) as described in the work of Cousins et al. (2020). Each of these constructs is briefly presented below.

#### Presenting

The way the object is introduced and presented may be influenced or even determined by environment, participant, and stimulus attributes (Table 2).

**Table 2.** Factors That Influence Object Presenting

Environmental attributes	Participant attributes	Stimulus attributes
Location	Gender	Shape
Number of people	Age	Size
Social context	Ethnicity	Weight
Cultural context	Level of dementia	Texture
Room temperature	Type of dementia	Surface characteristic
Room light	Person’s attitude to objects	Object history
Room noise	Previous experiences	Object role
Facilitator competencies	Person’s mood on the day^a^	Object meaning
Time stimuli presentation^a^	Physical and sensory impairment^a^	Smell
Seating arrangement^a^	—	Color^a^
Duration of session^a^	—	Density^a^

^a^Factors included following the consultations.

##### Environmental attributes

.— Environmental attributes include, for example, the location where the sessions are held; the number of people in the session; the social and cultural context; the level of temperature, noise, and light; the time of day of stimulus presentation, as well as the facilitator competencies. According to a fairly recent taxonomy (Cousins et al., 2020), facilitator competencies include training and skills, such as flexibility, empathy, practicality, intuition, tacit knowledge, knowledge of the objects used in the program, and having an engaging attitude.

##### Participant attributes

.— Relevant participant characteristics include aspects such as age, gender, level of dementia, physical and sensory abilities, and their likely previous experiences and familiarity with the items.

##### Stimulus attributes

.— Stimulus attributes include physical features of the material object, such as shape, size, weight, density, smell, and texture, as well as nonphysical or intangible aspects such as the object’s meaning, role, and history.

#### Receiving

The second component of object handling is the “receiving” stage, which refers to the moment when the participants interact with the material objects. This process is determined by the mutual intersection of material object, subject, and environment.

##### Environment–stimulus interaction

.— The physical spaces and social context in which object handling interventions are encountered can influence how the person engages with the items. For instance, a supportive and encouraging environment is more likely to be perceived by participants as a safe space to explore and engage with the objects (environment–stimulus interaction).

##### Participant–stimulus interactions

.— The interaction between the sensory, physical, and material characteristics of the objects and the person’s attributes will influence how the person reacts on receiving the object. Some stimuli may be more interesting for individuals than others. One example of a participant–stimulus interaction is the degree to which the person has shown a preference for this type of stimulus in the past. How the person receives and interacts with the object will also be affected by their individual characteristics such as cognitive and sensorial impairments, and their past interests and experiences, such as hobbies or work.

#### Responding

Receiving of the material object is followed by the participant’s response, which can be seen as complex patterns of emotions, cognitions, sensations, and interactions, that may be expressed through verbal and nonverbal communication (see Supplementary Table 4). Equally, manifesting no response is a perfectly legitimate way of responding.

#### Principles

Cousins et al. (2020) developed a taxonomy of the key components of arts interventions in dementia. One of the most important dimensions of this system was the concept of principles, a term used to identify the key components in generating an arts intervention. The list of eight principles derived by Cousins et al. (2020) was used to reexamine the papers reviewed, and those that are relevant were summarized in Supplementary Table 5.

### Limitations

Limitations of this study include the relatively narrow range of studies identified under the term of “object handling.” It may be that there are similar studies that have not used this specific term or the keywords used in the research strategy. To clarify the boundaries between object handling and other interventions in dementia care (e.g., occupational or reminiscence therapy) and to strengthen the review process, it would have been useful to summarize the characteristics of the studies using material objects which were excluded from the review.

The results of this review extend the initial definition of object handling, exploring the components and likely domains of the intervention action. Drawing on broader evidence provides a comprehensive overview of the concept and potential mechanisms affecting object handling outcomes. Although the object handling guidance was developed from papers included in the review, other papers identified through reference checks, and other key studies identified by professional stakeholders, there is the risk that relevant papers may have been missed.

A further limitation lies in the lack of geographic diversity of the studies included which may affect the generalization of our findings. Indeed, most of the studies reviewed are from the United Kingdom. Given the focus on heritage objects in the studies so far published, there is a need for more studies involving the handling of everyday material objects because these are by definition highly accessible and may have wider relevance for diverse populations.

### Recommendations for Future Research

The review highlighted that there are a relatively small number of studies of object handling in dementia. The studies varied considerably in their designs and methodologies. It is also important to note that heritage objects were used in most of the dementia-focused object handling studies. It is therefore important to bring together the published studies using object handling as their stated approach, with the aim of using more precise terminology and definitions, which will encourage consistency in study design and reporting.

Given the evidence above, further research is needed to examine and assess the complexity of object handling intervention, its components, and the mechanisms by which object handling may exert beneficial effects. Indeed, there is little evidence to inform how object handling interventions work, apart from the fairly recent publication by Camic et al. (2021) exploring the process underpinning the impact of object handling sessions on well-being in museum settings. Furthermore, there is a need for more studies involving the handling of everyday material objects because these are widely available and highly accessible. Everyday and familiar items can provide opportunities for storytelling, including spontaneous memory recall, and sharing experiences and emotions associated with the objects. In their new conceptual framework of “being in the moment”, Keady et al. (2022) suggest that recalling and revisiting past events enable individuals to reconnect and relive the “moment” as part of a continuum of moments moving forwards in time. The reminiscence effect enhanced by familiar items can therefore be seen as a means for creating a space for promoting connections, enjoyment, interactions, and engagement in the present.

Future studies could usefully apply theoretical rigor, for example, by using the principles that have been outlined in a empirically derived taxonomy of arts interventions (Cousins et al., 2020). Supplementary Table 5 indicates how the principles of Cousins et al. (2020) might be applied to object handling to develop future interventions.

Future research should focus on identifying the benefits and even negative impacts (Kinsey et al., 2021) of object handling sessions in people with dementia as well as care professionals and informal caregivers using qualitative methods that enable the capture of “in the moment” effects. However, future research may also benefit from mixed-methods and rigorous quantitative design, including randomized allocation, which could offer the opportunity to assess the effect size in relation to control group outcomes, lending further weight to the value of object handling as distinct to other, nonspecific factors such as the nature of the group or environmental setting. Although quantitative research is particularly useful to quantify intervention effects, qualitative methods such as video or creative methods (Fleetwood-Smith et al., 2021) enable capture of the sensory, emotional, and embodied experience of people with dementia with material objects, and positive effects that are likely to occur “in the moment” at verbal and nonverbal levels (Fleetwood-Smith et al., 2021; Tsekleves and Keady, 2021), providing further insights into the experience of handling and discussing material objects. Qualitative measures along with other objective measures such as noninvasive physiological measures such as skin conductance or cardiovascular response using new technologies and instruments could also provide valuable information about the experience of people with dementia, especially those with communication difficulties, participating in object handling sessions (Walker et al., 2021).

## Conclusion

This review, bringing together studies of object handling interventions, assesses the available evidence and explores the study designs and outcomes. Given the evidence outlined above, it can be stated that people with dementia may benefit from object handling interventions as a means of improving well-being, mood, and social inclusion. The results from this review have been used to build a better understanding of the factors involved in the process of object handling in people with dementia that can support implementation in health, social care, and community settings.

## Supplementary Material

igac043_suppl_Supplementary_Tables
