# Supplementary material for: Object Handling for People With Dementia: A Scoping Review and the Development of Intervention Guidance
Source: Innov Aging. 2022 Jun 13;6(5):igac043. doi: 10.1093/geroni/igac043 (PMC9331071; doi:10.1093/geroni/igac043)
Supplement: igac043_suppl_Supplementary_Tables [file igac043_suppl_supplementary_tables.docx]

Supplementary Table 1. Summary of search terms

| **Search** | **Terms** |
| --- | --- |
| #1 | object OR handling OR museum* OR objects |
| #2 | dement* OR alzheim* |
| #3 | mice OR rat |
|  | #1 AND #2 NOT #3 |

Supplementary Table 2. Characteristics of studies included

| **Study & Country** | **Study design & Setting** | **Sample** | **Mean/range age** | **Intervention** | **Target outcome & Measures** | **Results** |
| --- | --- | --- | --- | --- | --- | --- |
| Camic  et al. ^33^  UK | Mixed method design  Museum | 4 Persons with dementia  Mild to moderate  2 AD^a^  FTD^b^ | 69 ± 11.46 | Object handling  3 sessions  1 hour (plus 1h refreshment)  3 weeks | Subjective well-being  MMSE – 2 brief version;  clinical dementia rating  Video & audio-recorded  VAS^k^ - Canterbury  Wellbeing  Scale | Significant improvement in the Interested/Bored VAS^k^ subscale. Increase but not significant in all VAS^k^ items before and after the session, and pre- and post-intervention. Qualitative findings: facilitating conditions led to the active participation of group members, exploring objects. This promoted group collaboration. |
| Innes  et al. ^38^ UK | Mixed method design  Museums | 24 Persons with dementia  42% AD^a^  21% Mixed dementia  24 Caregivers | 62-94 | Object handling Storytelling  Visiting museum | Subjective well-being  Interviews  Mood questionnaires | Participants 'enjoyed' and 'valued' their participation. Mood questionnaires showed an increase in mood scores from pre- post session of 26% for people living with dementia and 20% for care partners. |
| Camic  et al. ^21^  UK | Quasi-experimental (non-randomised design)  Day centre & museum | 80 Persons with dementia  50 early  30 mild  37 AD^a^  24 VaD^j^  4 Mixed-types  2 HIV-related | 74.81 ± 7.6 | Object handling (h)^c^  1 session  60 min | Subjective well-being  Clinical Dementia Rating  Audio-recorded  VAS^k^ - Canterbury  Wellbeing  Scale | Significant improvement of well-being in both settings and stages of dementia, but higher effect in early-stage than mild. Younger participants benefit most from the intervention. |
| Griffiths  et al. ^34^  UK | Qualitative  Care home | 13 Persons with dementia  Mild to moderate  3 Care professionals & other staff | - | Object handling (h)^c^  6 sessions  6 weeks  120 min | Emotional responses  & engagement  Interview | Positive engagement, social interactions and emotions. |
| Hendriks  et al.^32^  NL | Quantitative  (cross-sectional observational design)  Museums | 72 Persons with dementia^j^  10 mild  27 moderate  13 severe  20 AD^a^  7 VaD^j^  2 FTD^b^  Other 14 | 81.33 ± 7.64 | Art viewing  Object  handling (h-m)^c d^    1 session  90 min | Engagement &  interactions  Observation Questionnaires  Assessment of Art Attributes | Active responsive and interactions in those with moderate levels of impairment.  Increasing responsive with objects compared to artworks. |
| Thomson  et al. ^36^  UK | Mixed method  Museums | 115 (not all participants with dementia)  Mild to moderate dementia | 65-94 | Art making  Art viewing  Object handling (h)^c^  10 sessions  10 weeks  120 min | Psychological  well-being  MWM-OA^h^  Participants’ diaries  Interview | Significant improvement in well-being. |
| Johnson  et al. ^35^  UK | Quasi-experimental (non-randomised  crossover  design)  Museum | 36 Persons with dementia^j^  Early to mild  3 MCI^f^  17 AD^a^  8 Mixed-types  5 FTD^b^  4 VaD^j^  30 caregivers | 74 ± 7.6  Persons with dementia^j^  66 ± 9.95 Caregiver | Object handling (h)^c^  Social activity (i.e. refreshment break)  Art viewing  1 session  115 min | Subjective well-being  Clinical Dementia Rating  Questionnaire  VAS^k^ - Canterbury  Wellbeing  Scale | Significant increase in  well-being following object handling and art viewing but not during social activity. The increase was significantly greater from object handling than art viewing. |
| Roe  et al. ^41^  UK | Qualitative  Museum | 17 participants  10 Care professionals  1 Caregiver | 75-92 | Object handling (h)^c^  Visiting museum  Art making  6 sessions  24 weeks  180 min | Subjective  well-being  Observation, interview  Field notes & session summary | Positive benefit reported on well-being, mood, social engagement and memories. |
| Ander  et al. ^40^  UK | Qualitative  Health care settings | 42 Persons with dementia  40 Neurological rehabilitation in/out patients  8 Healthcare staff | - | Object handling (h)^c^  1 - 8 sessions  over 1 - 16 weeks  (Participants took part in one or multiple sessions) | Emotions, feelings & life experiences  Interview  Observation  Field notes | Positive benefit reported on well-being, emotion, and participation. Stimulating social inclusion and new learning. |
| Thomson  et al. ^39^  UK | Mixed method  Hospital &  care home | 10 Persons with dementia  40 Neurological rehabilitation in/out patients  94 Oncology patients  14 Acute and elderly care  21 Control group | - | Object handling (h)^c^  Pictures (c.g.)^e^  1 session  40 min | Psychological  well-being, subjective well-being & happiness  PANAS^i^  VASs^k^ - wellness and happiness | Increasing wellness, happiness and positive mood in experimental group compared to control group. Negative mood decreased in both groups but no significant difference between groups were found. |
| Norberg  et al. ^37^  Sweden | Case study  Care home | 2 Persons with dementia  Severe | 87 & 83 | Object handling (m)^c^  Touch  Music  16 sessions  2 weeks  70 min | Physical & physiological responses  Observation, video & audio-recorded, pulse & respiration rate | Lower frequency of eye blinking, higher verbal reactions and pulse rate during music compared to objects handling and massage. |

*^a^(AD) Alzheimer’s Disease; ^b^(FTD) = Frontotemporal Dementia; ^c^(h) = heritage objects; ^d^(m) = modern objects; ^e^(c.g.) = control group; ^f^(MCI)* Mild*Cognitive Impairment;* *^g^(MMSE) = Mini Mental State Examination; ^h^(MWM-OA) = Museum Wellbeing Measure for Older Adults; ^i^(PANAS) = Positive and Negative Affect Scale; ^j^(VaD) = Vascular Dementia; ^k^(VAS) = Visual Analog Scales.*

Supplementary Table 3. Object handling material objects

| **Study & Country** | **Type of material objects** | **Material objects** |
| --- | --- | --- |
| Camic et al. ^33^  UK | Museum collection, other material objects and spices | Gomez boxes with objects from 19th -21st centuries, knitted neurons, prosthetic hand, C20th baby's feeding bottle, fisherman's floats, acupuncture teaching model, 19th century metal key, woven basket, sandalwood elephant, lucky iron fish, crystal slice, Yoruba twin figures, saltshaker, obsidian mirror, floor protector, turmeric, cloves, cinnamon, black pepper. |
| Innes et al. ^38^  UK | Heritage material objects | Hampton Court and Kew Palaces objects and artefacts. |
| Camic et al. ^21^  UK | Museum material objects | Objects included e.g., a tiger’s skull, fossilised seaweed, Victorian candle snuffer, preserved cotton bud, Stone Age New Zealand hand axe, Egyptian mummy wrapping sample, 19th-century biscuit tin, Islamic porcelain, Roman mosaic floor and Tunbridge Ware. |
| Griffiths et al. ^34^  UK | Heritage material objects and olfactory stimuli including spices | Objects associated with six themes: Daily Routine (sponge, hairbrush, shavers, poster, photos, toothpaste, Cremolia soap, talcum powder). Parenthood Feeding (bottle, feeding bowl, bibs, oral pacifier, baby soap, baby lotion, baby powder). Illness Chamber pots, thermometer, inhaler, hot water bottle, medicine tins, crushed aspirin, vapour rub). Childhood (children’s books, dolls, toy trolleys, owl puppet, spinning top, first aid kit, building blocks, leather satchel, TCP, Germolene, cough mixture, liquorice, crushed Parma Violets, crushed pear drops). Out on the Town (hats, scarves, ties, handbag, deodorant bottle, powder compact, floral perfumes, brilliantine hair cream). Christmas (tissue paper, Christmas mug, soap gift set, decorations, party hooters, bells, gift vouchers, Christmas cards, Boots catalogues, mince pies, cloves, cinnamon, candle). |
| Hendriks et al.^32^  NL | Museum material objects | The items ranged from historical objects to modern art and mounted animals. |
| Thomson et al. ^36^  UK | Museum material objects | Objects included museum collections such as anthropological items. |
| Johnson et al. ^35^  UK | Museum material objects | Objects used included for example Victorian carbolic soap, ancient Egyptian scarab stone, Iron Age axe head, geode, 19th-century African headdress rest, fossilized shark’s tooth, 18th-century tinderbox. |
| Roe et al. ^41^  UK | Museum and gallery material objects | Objects included such as boxes of insects, cotton heads in a box, a beetle, and tokens from Belle Vue Zoo. |
| Ander et al. ^40^  UK | Museum material objects | Objects comprised specimens from anthropology, archaeology, art, geology, and zoology collections (e.g., ammonite fossil, Neolithic axe head, elephant tooth section, coral specimen, Roman tile, and talc mineral) |
| Thomson et al. ^39^  UK | Museum material objects | Archaeological and ethnographic artefacts; etchings and printing plates; fossils, mineral samples, and natural history specimens (e.g., Egyptian eye amulet, painted pottery, fossil shark's tooth, turtle carapace, sodalite specimen). |
| Norberg et al. ^37^  Sweden | Everyday material objects | Objects presented were bread, wood, hay, soft soap, fur, camphor, yarn, tar. |

Supplementary Table 4. Object handling participants’ responses

| Emotional | Participants may experience one or likely a range of emotions, which define the subjective experience. Emotions such as ‘happiness’, ‘anger’, ‘sadness’ and ‘boredom’ might be expressed through the overt expression of emotion, or by non-verbal and/or physiological responses such as body language, sweaty palms, or a racing heartbeat. |
| --- | --- |
| Cognitive | Participants are actively engaged in meaning-making which occurs through interaction and communication with the group members or the facilitator. A variety of cognitive domains such as attention, sensory integration, memory, and executive function are typically involved during the object handling process. |
| Interaction | Objects can facilitate verbal and non-verbal communication. People may respond by talking about the stimulus with the facilitator or group members. However, people may react to the object non-verbally, through physical engagement, exploring and manipulating objects, or by using a bodily interaction, such as body posture, direction of gaze and gestures, without any explicit, verbal reference to what they mean. |
| Sensorial | Handling objects promotes the exploration of the items in a multi-sensory manner. For instance, a person can engage with the item using one or more of the five senses including visual, olfactory, and tactile sensory modalities. |

Supplementary Table 5. Descriptions and lay summaries of the principles (Cousins et al. ^53^) involved in object handling as an intervention in dementia

| **Principle description** | **Principle lay summary** |
| --- | --- |
| **Intellectual stimulation**: object handling promotes thinking, meaning-making, information transfer and new learning. | The object handling enables thinking. |
| **Therapeutic**: object handling facilitate symbolisation, affect, embodiment, well-being, identity, feelings of belonging, reminiscence, social and cultural inclusion. | The object handling can address the needs of people. |
| **Explorative**: object handling engages, stimulates, and triggers curiosity. | The object handling promotes investigative behaviours. |
| **Cognitively sensitive**: object handling can be adapted and modelled to the participants’ cognitive resources. | The object handling is sensitive to individual differences. |
| **Creative**: object handling provides the opportunity to think creatively about touch and objects. | The object handling allows imagination and creative process. |
| **Connection**: object handling can facilitate connection to the self and to others, enhancing group-building and encouraging interaction within the group. | The object handling enables social interactions. |
